# Supplementary material for: Improving physical activity, sedentary behaviour and sleep in COPD: perspectives of people with COPD and experts via a Delphi approach
Source: PeerJ. 2018 Apr 27;6:e4604. doi: 10.7717/peerj.4604 (PMC5926552; doi:10.7717/peerj.4604)
Supplement: Table S1 — NR, no response; RX, respondent number. [file peerj-06-4604-s001.docx]

**Table S1.** Participant feedback from pilot of Round 1 Delphi questionnaire with research team action.

|  | | | | | | |
| --- | --- | --- | --- | --- | --- | --- |
| **Survey question** | | **Feedback received from participants** | | | | **Research team action** |
|  |  | SA-COPD group  (n=4) | NL-COPD group  (n=6) | COPD-E group  (n=6) | Non-COPD-E group  (n=5) |  |
| 1. How long did it take to complete the survey [the Round 1 questionnaire]? | | | | | | |
|  | *0-10 min* | 0% | 50% | 80% | 60% | Advise participants that the survey should take less than 20 minutes |
|  | *10-15 min* | 75% | 17% | 20% | 40% |  |
|  | *15-20 min* | 25% | 33% | 0% | 0% |  |
|  | *20-30 min* | 0% | 0% | 0% | 0% |  |
|  | *>30 min* | 0% | 0% | 0% | 0% |  |
| 1. Were the questions easy to understand? | | | | | | |
|  | *n=, %yes* | 4 (100%) | 6 (100%) | 5 (100%) | 3 (60%) | Nil |
| 1. Which questions were difficult to understand and why? | | | | | | |
|  | *Verbatim response* | NR | NR | NR | R5: ‘Not sure exactly what you were looking for’ | Nil |
| 1. Was the open-ended question clear and easy to understand? | | | | | | |
|  | *n=, %yes* | 4 (100%) | 6 (100%) | 5 (100%) | 3 (60%) | Nil |
| 1. How could the open-ended question better be phrased? | | | | | | |
|  | *Verbatim response* | NR | NR | NR | R4: ‘I would suggest ‘what strategies’ ….’ | No changes made to question |
| 1. Is there any further information that was not covered in this survey that you feel would be useful/appropriate to collect? | | | | | | |
|  | *Verbatim response* | R1: ‘I really appreciated the program at The Repat which emphasised keeping active.’  R2: ‘I find it easier to breathe on cold days and nights if the air is kept a little warm.’  R4: ‘Other symptoms such as back pain and leg pain’ | R4: ‘Which activities do patients have’  R6: ‘no idea. For me only improvement of my saturation is important to be active more often and I wonder whether this questionnaire would contribute to it’ | R2: ‘…not really sure if this was the kind of response you were looking for. I find it very hard to generalise responses to these questions because as a clinician I take an individual approach that is a collaboration with the patient…’  R3: ‘…not clear what the objective is.’ | R4: ‘Do you need to know more about the barriers that stop people from being more active, so that appropriate strategies can be explored?’ | Nil |
| 1. Do you have any other feedback to provide which would help to develop this survey? | | | | | | |
|  | *Verbatim response* | R1: ‘I think if possible to keep weight down, that really helps. A positive attitude. Going north in the winter to minimise catching colds etc.’  R2: ‘I have been told my attitude and high motivation are enabling me to do twice as much as others with same lung capacity (38%)’ | R4: ‘I think there should be paid more attention to the daily time spending active’ | NR | R1: ‘…The questions were very open and so can elicit a broad range of responses.... which is probably what you are wanting.’  R2: ‘In general, the questions are quite broad. I appreciate that you might be interesting in minimising the extent to which you "lead" respondents down certain paths (e.g., are exercise programs useful, why?). However, you might consider offering some specificity while keeping it broad enough for open-ended responses…’ | Addition of question: In the last week on how many days did you participate in physical activities such as brisk walking, jogging, swimming, tennis or any other forms of exercise for at least 30 minutes? |
| NR: no response; RX: respondent number | | | | | | |
