# Supplementary material for: Improving physical activity, sedentary behaviour and sleep in COPD: perspectives of people with COPD and experts via a Delphi approach
Source: PeerJ. 2018 Apr 27;6:e4604. doi: 10.7717/peerj.4604 (PMC5926552; doi:10.7717/peerj.4604)
Supplement: Table S2 — COPD, chronic obstructive pulmonary disease; RX, respondent number. [file peerj-06-4604-s002.docx]

**Table S2.** Proposed amendments and action taken to allocation of items to themes by participants in Round 1b.

|  | | | | |
| --- | --- | --- | --- | --- |
| **Participant** | **Behaviour** | **Item/s to amend** | **Suggested amendment by participant** | **Action by research team** |
| South Australian COPD group | | | | |
| R6 | Sleep | ‘Don’t Know’ | [move to theme] Manage stress | Do not adopt proposed change; suggested changes are already covered with existing items and themes |
| R16 | Sedentary behaviour | Home exercise program | [move to theme] Managing co-existing problems and conditions | Do not adopt proposed change; suggested changes are already covered with existing items and themes |
| R8 | Sedentary behaviour | develop social contacts …Belonging to various clubs and groups is mentally stimulating | My words of “Develop social contacts” does not, in my opinion, equate with “Social Support”. Club membership does in some cases result in social support but it cannot be guaranteed because this depends usually on the kindness of the members and also to a certain extent on the social skills of the new member (except perhaps in the case of the Lung Net Club and other medically based clubs). I would prefer the heading “Social Interaction” | Change –other. Heading of theme ‘Social Support’ changed to ‘Social Support and Interactions’ |
|  |  | Attitude | I did however think that not enough was made of” Attitude”. A positive attitude does a lot to help all health problems  and also helps to maintain family support etc. | No change |
| Netherlands COPD group | | | | |
| R15 | Physical activity | Ben pas een maand bezig met een bewegings programma dus kan daar nog niet veel over zeggen | [ move to theme] Verbeteren van uithoudingsvermogen/fitness and Verbeteren/behouden van dagelijkse activiteiten | Do not adopt proposed change; suggested changes are already covered with existing items and themes |
| COPD expert group | | | | |
| R6 | Sedentary behaviour | Provide reasons not to be inactive | [move to theme] Understand patients fears concerns and expectations | Move participant’s response to the suggested existing theme |
| Non-COPD expert group | | | | |
| R5 | Physical activity | Encourage light-moderate activities like walking. | [move to theme] Self-monitoring and goal setting | Move participant’s response to the suggested existing theme |
|  | Sedentary behaviour | Break up sitting/lying down time regularly (active breaks) | [move to theme] Education: How to reduce sedentary behaviour | Move participant’s response to the suggested existing theme |
| R6 | Sedentary behaviour | Education on why it's important to reduce sitting time and practical / easy strategies to help. | The response should be split between two groups - Education on why it is important - in Education.  Practical and easy strategies to help in - How to reduce | Do not adopt proposed change; suggested changes are already covered with existing items and themes |
| R1 | Sedentary behaviour | A structured program with goal setting for lifestyle modification is important (e.g. so that the patient has specific aspects of their daily life that they are working to change). The changes need to be small enough that the patient has a high likelihood of success, and incremental so that enough change is achieved for real benefit. | I think the first sentence has been appropriately allocated to goal setting. Whereas I think the second sentence should be in "increase physical activity/fitness" | Do not adopt proposed change; suggested changes are already covered with existing items and themes |
| COPD: chronic obstructive pulmonary disease; RX: respondent number | | | | |
