# Supplementary material for: Improving physical activity, sedentary behaviour and sleep in COPD: perspectives of people with COPD and experts via a Delphi approach
Source: PeerJ. 2018 Apr 27;6:e4604. doi: 10.7717/peerj.4604 (PMC5926552; doi:10.7717/peerj.4604)
Supplement: Table S3 — (1) COPD, chronic obstructive pulmonary disease; Employ., Modify/understand employment commitments; GP, general practitioner; PA, physical activity. (2) CFE, concerns/fears/expectations; COPD, chronic obstructive pulmonary disease; E, Modify/understand employment commitments; MCC, Manage co-existing problems and conditions; MS, manage symptoms; PA, physical activity; PE, Manage/understand the physical environment; SE, self-efficacy; SM, Self-monitoring (3) CPAP, continuous positive airway pressure; PA, physical activity; NS, Non-specific; SM/GS, Self-monitoring/goal setting Results from 9-point Likert scale where 1 = unimportant and 9 = very important. [file peerj-06-4604-s003.docx]

| **Table S3:**  What is important to be more active and participate in more every day activities - group average Likert-score for each item by theme | | | | | | | | | | | | | | | | | |
| --- | --- | --- | --- | --- | --- | --- | --- | --- | --- | --- | --- | --- | --- | --- | --- | --- | --- |
| **Theme** | **Item** | **COPD-E** | | | | **Non-COPD-E** | | | | **SA-COPD** | | | | **NL-COPD** | | | |
|  |  | Median (IQR) | | Mean (±SD) | | Median (IQR) | | Mean (±SD) | | Median (IQR) | | Mean (±SD) | | Median (IQR) | | Mean (±SD) | |
| **Accessible / Affordable exercise facilities** | to have access to an exercise program with low out of pocket costs | 7.5 | (5.75-9) | 7.08 | ±1.98 | 7 | (7-8.5) | 7.64 | ±1.03 | 9 | (8-9) | 8.25 | ±1.07 | 9 | (8-9) | 8.29 | ±1.38 |
|  | to have access to an exercise program that is free | 7 | (6-8) | 6.75 | ±2.18 | 7 | (6-8) | 6.91 | ±1.58 | 8.5 | (8-9) | 8.00 | ±1.64 | 9 | (9-9) | 8.50 | ±1.34 |
|  | to have more affordable community gyms | 7 | (5.75-8) | 6.25 | ±2.49 | 7 | (6.5-7.5) | 6.91 | ±1.30 | 8 | (7.75-9) | 8.04 | ±1.16 | 9 | (8-9) | 8.50 | ±0.85 |
|  | to have community gyms close to home | 7 | (6.75-7.25) | 7.00 | ±1.28 | 7 | (6-8) | 6.91 | ±1.58 | 8 | (7-9) | 7.63 | ±1.86 | 9 | (8-9) | 8.14 | ±1.29 |
|  | to have more gyms specific for people with lung conditions to attend | 6.5 | (5-7) | 6.25 | ±1.60 | 5 | (3.5-7) | 5.09 | ±2.66 | 8 | (7-9) | 7.33 | ±2.37 | 9 | (8-9) | 8.14 | ±1.70 |
| **Behaviour change / autonomy / self-efficacy** | to build confidence and develop skills to cope with symptoms and activities of daily living | 8 | (8-9) | 8.31 | ±0.75 | 8 | (8-9) | 8.09 | ±0.94 | 9 | (8-9) | 8.29 | ±1.23 | 9 | (8-9) | 8.29 | ±1.20 |
|  | to develop skills to change behaviour | 9 | (8-9) | 8.31 | ±0.85 | 8 | (8-9) | 8.18 | ±0.98 | 8 | (7.75-9) | 8.00 | ±1.29 | 9 | (8-9) | 8.36 | ±1.01 |
|  | to feel in control and empowered | 8 | (7-9) | 7.85 | ±1.21 | 8 | (7.5-9) | 8.00 | ±1.00 | 9 | (8-9) | 8.08 | ±1.64 | 9 | (7.25-9) | 8.14 | ±1.35 |
|  | to be taught how to regulate what they are doing to minimise pain, discomfort etc. | 7 | (7-9) | 7.69 | ±1.03 | 8 | (7-9) | 7.82 | ±1.17 | 8.5 | (8-9) | 8.29 | ±0.95 | 9 | (8-9) | 8.36 | ±1.15 |
|  | to be encouraged by health care providers to persevere through difficult periods (e.g. when they do not feel well or in bad weather) | 8 | (7-8) | 7.46 | ±1.27 | 7 | (7-8) | 7.45 | ±0.93 | 8 | (7-9) | 7.92 | ±1.10 | 9 | (8-9) | 8.43 | ±0.94 |
|  | to be encouraged by health care providers to seek education and information | 8 | (7-8) | 7.54 | ±0.97 | 7 | (7-8) | 7.09 | ±1.30 | 8 | (7-9) | 7.83 | ±1.49 | 9 | (8-9) | 8.43 | ±0.94 |
|  | to be provided with a written treatment plan including information about how to manage lung conditions based on symptom variation | 7 | (6-8) | 7.31 | ±1.18 | 7 | (6-8) | 6.82 | ±1.33 | 8 | (7-9) | 8.00 | ±0.98 | 9 | (8.25-9) | 8.50 | ±0.94 |
|  | to address and create thoughts that are helpful towards activity | 7 | (7-8) | 7.46 | ±1.05 | 7 | (7-7.5) | 7.18 | ±1.17 | 8 | (7-9) | 7.67 | ±1.58 | 9 | (8-9) | 8.14 | ±1.51 |
| **Education** | to be educated about acute COPD exacerbations (flare-up of symptoms) | 8 | (8-9) | 8.00 | ±0.91 | 8 | (7-8.5) | 7.73 | ±1.19 | 9 | (8-9) | 8.42 | ±0.83 | 9 | (8-9) | 8.21 | ±1.19 |
|  | to be provided with tailored and personalised advice on how to become more active | 8 | (8-9) | 8.23 | ±0.73 | 8 | (7-9) | 8.00 | ±1.10 | 8 | (7-8.25) | 7.75 | ±1.03 | 9 | (8-9) | 8.21 | ±1.25 |
|  | to be taught and offered breathing exercises (e.g. pursed lip breathing, diaphragmatic breathing, or yoga) if they experience breathlessness that limits physical activity | 8 | (7-8) | 7.38 | ±1.33 | 7 | (6.5-8) | 7.27 | ±1.01 | 9 | (8-9) | 8.38 | ±0.88 | 9 | (9-9) | 8.64 | ±0.84 |
|  | to be provided with education and counselling around the benefits of physical activity, including light intensity physical activity (e.g. house-hold chores), not just moderate to vigorous intensity physical activity (e.g. exercise walking, cycling) | 8 | (7-8) | 7.62 | ±0.96 | 8 | (7-8.5) | 7.45 | ±1.51 | 9 | (7-9) | 8.17 | ±1.05 | 8.5 | (8-9) | 8.07 | ±1.38 |
|  | to be taught how to manage stress and anxiety | 8 | (7-8) | 7.69 | ±1.11 | 7 | (6-8) | 6.73 | ±1.19 | 9 | (8-9) | 8.50 | ±0.66 | 9 | (7.25-9) | 8.07 | ±1.33 |
|  | to be provided with good mental health education, linking activity with personal values and beliefs | 8 | (7-8) | 7.85 | ±0.90 | 6 | (6-7.5) | 6.55 | ±1.13 | 8 | (7-9) | 7.88 | ±1.08 | 9 | (8-9) | 8.21 | ±1.25 |
|  | to be educated on what being active means | 7 | (7-8) | 7.23 | ±1.09 | 7 | (6.5-7.5) | 7.09 | ±1.22 | 7 | (7-8) | 7.17 | ±1.58 | 8.5 | (8-9) | 8.07 | ±1.27 |
| **Enjoyment** | to be helped to find activities that are enjoyable and not stressful | 7.5 | (6.75-8.25) | 7.42 | ±1.31 | 8 | (7-9) | 7.91 | ±1.04 | 8 | (6.75-9) | 7.42 | ±2.21 | 8.5 | (8-9) | 8.14 | ±1.10 |
|  | to be taught to assess and adjust their exercise/activity intensity | 8 | (7-8.25) | 7.75 | ±0.97 | 8 | (7-9) | 7.91 | ±1.14 | 8 | (7-9) | 7.67 | ±1.86 | 8 | (5.5-9) | 7.36 | ±2.02 |
|  | to be offered an extensive choice of activities | 7 | (6-9) | 6.83 | ±2.33 | 8 | (7-9) | 7.55 | ±1.51 | 8 | (7-9) | 7.25 | ±1.94 | 8.5 | (8-9) | 7.86 | ±1.61 |
|  | to keep involved in sports in alternative ways (e.g. being a tea lady instead of playing) | 7 | (4.75-7) | 5.92 | ±1.98 | 6 | (5-8) | 6.64 | ±1.69 | 7 | (5.75-8.25) | 6.83 | ±1.99 | 8 | (8-9) | 8.00 | ±1.36 |
|  | to have a dog to walk | 5 | (2.5-6.25) | 4.42 | ±2.54 | 5 | (5-6.5) | 5.18 | ±2.23 | 5.5 | (2-8) | 5.13 | ±3.03 | 8.5 | (8-9) | 8.14 | ±1.17 |
| **Increase PA/fitness** | to keep involved in daily activities including indoor activities (e.g. cleaning, shopping, washing and other house-hold chores) and/or outdoor activities (e.g. gardening, renovating, wood work) | 8 | (8-9) | 8.15 | ±0.80 | 8 | (8-9) | 8.27 | ±0.79 | 9 | (8-9) | 8.33 | ±0.96 | 9 | (8-9) | 8.36 | ±1.01 |
|  | to participate in regular exercise (e.g. cycling or daily walk or continuing at the gym) | 9 | (8-9) | 8.15 | ±1.14 | 8 | (8-9) | 8.27 | ±0.65 | 9 | (8-9) | 8.46 | ±0.72 | 8.5 | (8-9) | 8.21 | ±0.97 |
|  | to have sufficient fitness to stay active long-term | 8 | (8-9) | 8.08 | ±0.76 | 7 | (6.5-8) | 7.09 | ±1.38 | 9 | (8-9) | 8.54 | ±0.66 | 9 | (8.25-9) | 8.57 | ±0.76 |
|  | to continue with the exercise program which they have been participating in | 7 | (6-9) | 7.46 | ±1.45 | 8 | (7-8.5) | 7.73 | ±1.19 | 9 | (8-9) | 8.42 | ±0.72 | 9 | (8-9) | 8.43 | ±0.76 |
|  | to do endurance exercises (i.e. exercising at a lower level for longer periods of time) | 8 | (7-8) | 7.38 | ±1.19 | 7 | (7-8) | 7.27 | ±1.10 | 8 | (7-9) | 7.46 | ±1.74 | 8 | (7.25-9) | 7.71 | ±1.77 |
|  | to be encouraged by health care providers to be active every day, even in bad weather and on days which they do not feel so well | 8 | (7-8) | 7.38 | ±1.33 | 7 | (7-8.5) | 7.55 | ±1.21 | 8 | (7-9) | 7.79 | ±1.28 | 9 | (8-9) | 8.21 | ±1.19 |
|  | to exercise at home under the supervision of volunteers | 6 | (5-7) | 5.69 | ±1.97 | 6 | (5-6) | 5.91 | ±1.45 | 7 | (6-8) | 6.71 | ±1.94 | 9 | (8-9) | 8.29 | ±1.38 |
|  | to continue to do physical labour (e.g. helping at the farm, renovating, yard work) | 7 | (5-7) | 5.77 | ±2.20 | 8 | (6-9) | 7.18 | ±1.99 | 7 | (5.75-9) | 6.96 | ±2.10 | 8 | (7-9) | 7.57 | ±1.45 |
| **Manage co-existing problems / conditions** | to be able to manage other co-existing medical conditions (e.g. pain, heart problems, conditions which affect mobility) and have rehabilitation attention after surgeries such as hip replacements | 9 | (7.75-9) | 8.33 | ±0.89 | 8 | (7.5-9) | 8.00 | ±1.00 | 9 | (8-9) | 8.67 | ±0.56 | 7.5 | (7-8.75) | 7.00 | ±2.29 |
|  | to keep the whole body in the best possible condition (e.g. joints, muscle strength, aerobic fitness, flexibility, and balance) | 8 | (7-9) | 8.00 | ±0.85 | 8 | (7-8.5) | 7.82 | ±0.98 | 9 | (8-9) | 8.58 | ±0.72 | 8 | (6-9) | 7.07 | ±2.30 |
|  | to have support with quitting smoking | 8 | (7-9) | 7.33 | ±2.23 | 8 | (7.5-9) | 8.09 | ±1.04 | 9 | (7.5-9) | 7.25 | ±2.92 | 7 | (6.25-8.75) | 7.21 | ±1.53 |
|  | to lose weight | 6.5 | (5-7) | 6.00 | ±1.95 | 7 | (6-8) | 6.91 | ±1.70 | 8 | (5-9) | 6.50 | ±2.87 | 8 | (7-8.75) | 7.71 | ±1.20 |
| **Manage Symptoms** | to have symptoms of breathlessness and fatigue managed | 8 | (8-9) | 8.00 | ±1.15 | 8 | (7.5-9) | 8.00 | ±1.00 | 9 | (9-9) | 8.75 | ±0.53 | 8.5 | (7.25-9) | 8.00 | ±1.30 |
|  | to be provided with appropriate drug treatment, i.e. preventative puffers | 8 | (7-9) | 7.92 | ±1.12 | 8 | (6.5-8.5) | 7.64 | ±1.21 | 9 | (9-9) | 8.71 | ±0.69 | 8.5 | (7-9) | 7.79 | ±1.53 |
|  | to be provided with oxygen during exercise and activities of daily living if oxygen levels decrease | 7 | (5-7) | 6.15 | ±2.15 | 7 | (6-8) | 6.91 | ±1.30 | 9 | (6-9) | 7.54 | ±2.21 | 9 | (8-9) | 8.29 | ±0.99 |
| **Employ.** | to continue to work | 7 | (5.75-8) | 6.17 | ±2.62 | 7 | (6.5-8) | 7.27 | ±1.27 | 8 | (7-9) | 7.29 | ±2.48 | 9 | (8-9) | 7.93 | ±2.20 |
|  | to have less physical exhausting work | 6 | (5-7) | 5.92 | ±2.19 | 6 | (5-6) | 5.64 | ±1.29 | 8 | (6.75-9) | 6.96 | ±2.48 | 9 | (8-9) | 8.29 | ±0.99 |
|  | to have less hours at paid employment | 5 | (4-6) | 4.67 | ±2.50 | 5 | (3.5-5) | 4.36 | ±2.06 | 6 | (2.75-7) | 5.25 | ±2.83 | 9 | (8-9) | 8.36 | ±0.93 |
| **Modify / understand the physical environment** | to have flat, well surfaced footpaths for walking in the neighbourhood | 7 | (6-8) | 6.85 | ±2.08 | 7 | (7-8) | 7.55 | ±0.93 | 8 | (7-9) | 7.35 | ±2.06 | 8 | (7-8.75) | 7.64 | ±1.39 |
|  | to be able to easily practice physical activity in their neighbourhood | 8 | (7-9) | 7.31 | ±2.14 | 8 | (7.5-9) | 8.18 | ±0.87 | 8 | (7-9) | 7.21 | ±2.17 | 8.5 | (7.25-9) | 8.00 | ±1.30 |
|  | to consider the built environment (roads, pathways) and green spaces (parks) | 7 | (6-8) | 6.92 | ±2.06 | 7 | (7-9) | 7.82 | ±0.98 | 8 | (5.75-9) | 7.00 | ±2.47 | 8.5 | (8-9) | 8.21 | ±0.97 |
|  | to have public benches in public spaces to enable rests | 8 | (6-9) | 7.00 | ±2.27 | 8 | (7-8) | 7.73 | ±0.79 | 8 | (7-9) | 7.33 | ±2.12 | 8 | (7-8.75) | 7.57 | ±1.34 |
|  | to monitor and plan activity according to the weather | 7 | (6-8) | 6.62 | ±1.94 | 7 | (6.5-8.5) | 7.36 | ±1.36 | 7 | (6.75-9) | 7.25 | ±1.57 | 7 | (5.25-8) | 6.86 | ±1.66 |
|  | to be provided with a mobile seat, to enable rests whenever/wherever needed | 7 | (7-8) | 6.69 | ±1.97 | 6 | (6-7.5) | 6.55 | ±1.69 | 7 | (4-8) | 5.96 | ±2.87 | 8.5 | (7.25-9) | 7.93 | ±1.44 |
| **Non-Specific** | to consistently use their medication as instructed by doctor/nurses | 8 | (7.75-9) | 8.08 | ±1.00 | 9 | (7.5-9) | 8.09 | ±1.22 | 9 | (9-9) | 8.75 | ±0.53 | 8.5 | (8-9) | 8.21 | ±0.97 |
|  | to be resilient after set backs | 8 | (7.75-9) | 7.75 | ±1.96 | 8 | (7-8.5) | 7.64 | ±1.12 | 9 | (8-9) | 8.67 | ±0.48 | 8 | (7.25-9) | 7.93 | ±1.27 |
|  | to be compliant with medical care | 8 | (7-8) | 7.83 | ±0.72 | 8 | (7-9) | 7.82 | ±1.17 | 9 | (8-9) | 8.67 | ±0.48 | 8 | (7-8.75) | 7.64 | ±1.22 |
|  | to be accepting of disease process | 7.5 | (7-8) | 7.33 | ±1.37 | 7 | (7-8) | 7.27 | ±0.90 | 9 | (8-9) | 8.08 | ±1.72 | 8.5 | (8-9) | 8.07 | ±1.27 |
| **Professional support** | to have a GP and/or medical specialist who is supportive of lifestyle management | 8 | (7-9) | 8.08 | ±0.86 | 8 | (7.5-8.5) | 7.91 | ±0.94 | 9 | (8-9) | 8.42 | ±0.72 | 9 | (9-9) | 8.43 | ±1.28 |
|  | to participate in pulmonary rehabilitation programs | 8 | (8-9) | 8.23 | ±0.83 | 7 | (7-8) | 7.36 | ±1.03 | 9 | (8-9) | 8.46 | ±0.88 | 9 | (8-9) | 8.57 | ±0.65 |
|  | to be managed and supported long-term by a multidisciplinary team (doctors, nurses, physiotherapists etc.) | 9 | (7-9) | 8.08 | ±1.12 | 7 | (6-9) | 7.45 | ±1.37 | 8.5 | (8-9) | 7.75 | ±2.23 | 9 | (8-9) | 8.36 | ±1.15 |
|  | to be provided with a realistic activity program | 8 | (8-9) | 8.31 | ±0.63 | 8 | (8-9) | 8.18 | ±0.75 | 8 | (7.75-9) | 7.96 | ±1.68 | 8 | (6.25-8) | 7.07 | ±1.86 |
|  | to have pulmonary rehabilitation programs which focus on integrating physical activity routines into daily life, including activates such as walking, gardening and house-hold activities | 8 | (8-9) | 7.92 | ±1.12 | 8 | (7-9) | 7.91 | ±1.22 | 9 | (7.75-9) | 8.04 | ±1.71 | 8 | (8-9) | 7.93 | ±1.33 |
|  | to continue training after the completion of a pulmonary rehabilitation program at home unsupervised, with weekly or monthly supervised sessions | 9 | (7-9) | 8.23 | ±0.93 | 8 | (8-9) | 8.27 | ±0.79 | 8 | (7.75-9) | 8.17 | ±0.92 | 8 | (7-8.75) | 7.64 | ±1.28 |
|  | to be followed up after an acute COPD exacerbation (flare up of symptoms) | 8 | (7-9) | 8.08 | ±0.86 | 8 | (7-8.5) | 7.82 | ±0.87 | 9 | (8-9) | 8.33 | ±0.92 | 8 | (8-9) | 8.07 | ±0.92 |
|  | to be taught about medical problems and symptoms, and how to deal with them | 8 | (7-8) | 7.85 | ±0.69 | 7 | (7-8) | 7.45 | ±1.13 | 9 | (8-9) | 8.46 | ±0.78 | 8 | (8-9) | 7.93 | ±1.21 |
|  | to be offered regular follow-up appointments with structured content, including assessment of physical activity and symptoms | 8 | (7-9) | 7.85 | ±0.99 | 8 | (6.5-8) | 7.45 | ±1.13 | 8.5 | (7.75-9) | 7.75 | ±1.98 | 9 | (8-9) | 8.43 | ±0.94 |
|  | to be provided with advice, encouragement and motivation from health care providers around participation in daily physical activity | 8 | (7-8) | 7.69 | ±0.85 | 7 | (7-8.5) | 7.55 | ±1.21 | 8 | (7-9) | 7.58 | ±1.35 | 8 | (7.25-9) | 8.00 | ±1.11 |
|  | to have more supervised exercise programs (by trained physiotherapists, exercise physiologists, nurses etc.) such as pulmonary rehabilitation, in a home or outpatient setting | 8 | (7-9) | 7.85 | ±1.07 | 7 | (6.5-7.5) | 6.91 | ±1.22 | 8 | (6.75-9) | 7.33 | ±2.01 | 9 | (9-9) | 8.71 | ±0.61 |
|  | to participate in interventions which include counselling to increase motivation toward changing behaviours for health (motivational -interviewing) | 8 | (7-8) | 7.46 | ±1.27 | 7 | (7-8) | 7.55 | ±0.82 | 8 | (7-9) | 7.29 | ±2.26 | 8 | (8-9) | 7.86 | ±1.56 |
|  | to be provided with tips to conserve energy and relevant equipment such as walkers/canes by physiotherapists and occupational therapists | 8 | (7-9) | 7.62 | ±1.26 | 6 | (6-7) | 6.55 | ±1.21 | 8 | (7-9) | 7.38 | ±1.95 | 9 | (7-9) | 7.86 | ±1.56 |
|  | to be able to easily contact and network with health-care providers to maintain motivation for exercise | 8 | (7-9) | 7.85 | ±1.07 | 8 | (6.5-8.5) | 7.45 | ±1.37 | 8 | (7-9) | 7.63 | ±1.24 | 5.5 | (4-7) | 4.93 | ±2.53 |
|  | to be provided with reminders, messages and prompts for emotional support | 7 | (7-8) | 7.00 | ±1.47 | 7 | (6-9) | 7.18 | ±1.60 | 7 | (5.75-8.25) | 6.63 | ±2.32 | 8.5 | (7.25-9) | 7.93 | ±1.38 |
| **Self-monitor / goal setting** | to be helped to structure their daily life to engage in light to moderate intensity physical activities such as walking in the neighbourhood, to and from shops/the bus etc. | 8 | (7-9) | 7.67 | ±1.30 | 9 | (7-9) | 8.18 | ±0.98 | 8.5 | (7-9) | 7.88 | ±1.78 | 9 | (8-9) | 8.21 | ±1.19 |
|  | to be provided with individualised, realistic and achievable goals and action plans for daily life activities | 8 | (7-9) | 7.83 | ±1.34 | 8 | (7.5-9) | 8.18 | ±0.87 | 8.5 | (7-9) | 7.92 | ±1.41 | 8 | (8-9) | 7.93 | ±1.59 |
|  | to be provided with positive feedback | 8 | (7.75-8.25) | 7.83 | ±1.03 | 7 | (7-8.5) | 7.45 | ±1.29 | 8 | (7-9) | 7.54 | ±1.89 | 9 | (8-9) | 8.07 | ±1.49 |
| **Social support** | to have supportive family members and have loved ones involved in their care | 8.5 | (7.75-9) | 8.08 | ±1.24 | 8 | (7-9) | 7.82 | ±1.40 | 8.5 | (8-9) | 7.88 | ±1.68 | 8 | (8-9) | 7.86 | ±1.66 |
|  | to have social support | 8 | (7-9) | 7.83 | ±1.19 | 8 | (7.5-9) | 8.18 | ±0.87 | 8 | (7-9) | 7.71 | ±1.55 | 8.5 | (8-9) | 8.14 | ±1.17 |
|  | to take part in social activities (e.g. meeting friends for lunch and going to the cinema) | 8 | (7-8) | 7.67 | ±1.07 | 8 | (6.5-8) | 7.27 | ±1.42 | 8 | (7-9) | 7.58 | ±1.67 | 8.5 | (8-9) | 8.14 | ±1.23 |
|  | to help each other and have formal (i.e. organised group sessions) and informal (i.e. walking buddies) peer support networks | 7.5 | (6-9) | 7.33 | ±1.56 | 8 | (7-8.5) | 7.91 | ±0.83 | 7.5 | (7-8) | 7.25 | ±1.51 | 8 | (8-9) | 8.00 | ±1.24 |
| **Understand patients concerns / fears / expectations** | to have self-motivation | 9 | (8-9) | 8.50 | ±0.67 | 8 | (8-8.5) | 8.09 | ±0.70 | 9 | (8-9) | 8.58 | ±0.78 | 8 | (7-8.75) | 7.71 | ±1.20 |
|  | to be educated on how to deal with breathlessness during exercise | 8 | (7-9) | 7.75 | ±1.22 | 8 | (7-9) | 8.00 | ±1.10 | 9 | (9-9) | 8.63 | ±0.77 | 8 | (8-9) | 8.14 | ±1.10 |
|  | to overcome fear of breathlessness | 8 | (7-9) | 8.08 | ±0.90 | 8 | (7-8.5) | 7.73 | ±1.01 | 9 | (8.75-9) | 8.58 | ±0.83 | 8 | (7.25-9) | 7.86 | ±1.23 |
|  | to be encouraged and reassured by health care providers that performing physical activity to the point of breathlessness is safe and not harmful, and over time, will increase their exercise abilities | 8.5 | (8-9) | 8.33 | ±0.78 | 9 | (7-9) | 8.09 | ±1.14 | 9 | (8-9) | 8.33 | ±0.96 | 8 | (7.25-8.75) | 7.71 | ±1.33 |
|  | to be less breathless | 8 | (7-9) | 7.75 | ±1.22 | 7 | (5.5-7.5) | 6.45 | ±1.37 | 9 | (8-9) | 8.50 | ±1.10 | 8 | (8-9) | 8.21 | ±0.89 |
|  | to reduce expectations that staying active causes pain, discomfort or breathlessness | 8 | (7-8.25) | 7.67 | ±1.15 | 7 | (6.5-8) | 7.27 | ±1.27 | 8 | (7-9) | 7.79 | ±1.18 | 8 | (8-9) | 8.00 | ±1.11 |
|  | to manage fears of the outdoors and getting infections/the cold | 8 | (7-9) | 7.83 | ±1.19 | 8 | (6.5-8.5) | 7.45 | ±1.37 | 8 | (7-9) | 7.75 | ±1.82 | 8 | (8-8.75) | 7.64 | ±1.55 |
|  | to be able to control their breathing better and do things without getting breathless | 8 | (7-8.25) | 7.58 | ±1.24 | 7 | (6-7.5) | 6.82 | ±1.25 | 9 | (8.75-9) | 8.63 | ±0.71 | 8 | (8-9) | 7.57 | ±2.03 |
| COPD: chronic obstructive pulmonary disease; Employ.: Modify / understand employment commitments; GP: general practitioner; PA: physical activity.  Results from 9-point Likert scale where 1 = unimportant and 9 = very important | | | | | | | | | | | | | | | | | |

| **Table S3:** What is important to spend less time sitting and lying - Group average Likert-score for each item by theme | | | | | | | | | | | | | | | | | |
| --- | --- | --- | --- | --- | --- | --- | --- | --- | --- | --- | --- | --- | --- | --- | --- | --- | --- |
| **Theme** | **Item** | **COPD-E** | | | | **Non-COPD-E** | | | | **SA-COPD** | | | | **NL-COPD** | | | |
|  |  | Median (IQR) | | Mean (±SD) | | Median (IQR) | | Mean (±SD) | | Median (IQR) | | Mean (±SD) | | Median (IQR) | | Mean (±SD) | |
| **Behaviour change / autonomy / SE** | to be encouraged to create positive thoughts towards being active | 8 | (7-9) | 8.00 | ±1.04 | 7 | (7-8) | 7.09 | ±1.30 | 9 | (8-9) | 8.26 | ±0.92 | 8.5 | (8-9) | 8.21 | ±1.12 |
|  | to be helped to develop self-confidence with managing symptoms such as breathlessness and fatigue | 8 | (7-9) | 8.00 | ±0.95 | 7 | (7-8) | 7.27 | ±1.01 | 9 | (8-9) | 8.39 | ±0.89 | 8 | (7-9) | 7.71 | ±1.27 |
|  | to improve their self-confidence to be able to change health behaviours | 8 | (7-9) | 7.92 | ±0.90 | 7 | (7-8) | 7.09 | ±1.30 | 9 | (8-9) | 8.26 | ±1.01 | 8.5 | (8-9) | 8.07 | ±1.27 |
| **Education** | to be made aware that the performance of daily activities (i.e. walking, standing, house-hold activities) will contribute to a more active lifestyle even if performed in brief episodes | 8 | (8-9) | 8.17 | ±0.94 | 7 | (7-8) | 7.55 | ±1.13 | 8.5 | (7-9) | 8.21 | ±0.88 | 9 | (8.25-9) | 8.64 | ±0.63 |
|  | to be made aware of and educated about the benefits of light-intensity activity (e.g. house-hold chores), not just moderate to vigorous intensity physical activity (exercise walking, cycling) | 8 | (7.75-9) | 8.00 | ±0.95 | 8 | (7-8.5) | 7.73 | ±0.90 | 8 | (7-9) | 8.13 | ±0.85 | 8 | (8-9) | 8.21 | ±0.80 |
|  | to be aware of the benefits of being active versus the consequences of being inactive | 8 | (7-8.25) | 7.75 | ±1.06 | 7 | (6.5-8) | 7.36 | ±1.12 | 9 | (8-9) | 8.42 | ±0.83 | 7.5 | (7-9) | 7.93 | ±1.00 |
|  | to be encouraged to and educated on how to structure their daily life to break up prolonged sitting time with regular activity breaks (e.g. engaging in small and easy activities such as walking to the bathroom/kitchen once per hour) | 8 | (8-9) | 8.08 | ±0.90 | 8 | (7-8) | 7.64 | ±0.92 | 8 | (7-9) | 7.42 | ±1.89 | 9 | (8-9) | 8.43 | ±0.76 |
|  | to be provided good mental health education, linking activity with personal values and beliefs | 8 | (7-8.25) | 7.75 | ±0.97 | 7 | (6-7.5) | 6.82 | ±1.47 | 8 | (7-9) | 7.96 | ±1.16 | 8 | (7.25-9) | 7.93 | ±1.14 |
|  | and their loved ones to be educated and counselled | 8 | (6.75-8.25) | 6.92 | ±2.68 | 7 | (6-7) | 6.82 | ±0.98 | 7.5 | (6-9) | 7.13 | ±1.90 | 9 | (8-9) | 8.50 | ±0.76 |
| **Enjoyment** | to find activities of interest | 8 | (7-9) | 7.92 | ±0.90 | 8 | (8-8.5) | 8.18 | ±0.60 | 8 | (7.5-9) | 7.74 | ±1.91 | 8 | (6.25-9) | 7.64 | ±1.55 |
|  | be provided with advice on enjoyable, meaningful alternatives to sitting and lying such as hobbies, scheduled activities and a social life | 7.5 | (6.75-8) | 7.25 | ±1.14 | 8 | (7-8.5) | 7.91 | ±0.83 | 8 | (7-9) | 7.52 | ±1.56 | 8 | (8-9) | 8.21 | ±0.80 |
|  | to have a dog to walk | 5 | (2.5-6.25) | 4.50 | ±2.50 | 5 | (5-5.5) | 4.91 | ±2.07 | 6 | (2.5-8) | 5.00 | ±3.07 | 8 | (8-9) | 7.93 | ±1.21 |
| **Increase PA / fitness** | to increase their fitness and physical capacity | 8 | (7.75-8) | 7.92 | ±0.67 | 7 | (6-8) | 6.55 | ±1.57 | 8 | (7.5-9) | 8.04 | ±1.22 | 9 | (8-9) | 8.21 | ±1.12 |
|  | to do endurance exercises (i.e. exercising at a lower level for a longer period of time) | 8 | (7-8.25) | 7.58 | ±1.38 | 6 | (5.5-7) | 6.18 | ±1.17 | 7 | (6.5-9) | 7.13 | ±2.05 | 8 | (7-9) | 7.71 | ±1.38 |
|  | to go to the gym regularly | 6.5 | (5.75-8) | 6.50 | ±1.68 | 5 | (5-6.5) | 5.55 | ±2.11 | 7 | (5-8.5) | 6.39 | ±2.41 | 8 | (7-9) | 7.79 | ±1.31 |
| **Increase / maintain daily activities** | to remain as independent as possible; live in their own home and keep involved in house-work (e.g. cooking, doing the dishes etc.) and outdoor activities (gardening etc.) | 8 | (8-9) | 8.17 | ±0.72 | 8 | (7.5-9) | 8.00 | ±1.00 | 9 | (8.75-9) | 8.75 | ±0.44 | 9 | (8-9) | 8.36 | ±0.84 |
|  | to increase time spent in light intensity physical activities (e.g. house-hold chores, standing, light walking) so it is the same as time spent sedentary (sitting, lying). | 8 | (7.75-8.25) | 8.00 | ±0.74 | 8 | (6.5-8) | 7.36 | ±1.29 | 8 | (8-9) | 8.17 | ±0.96 | 9 | (8-9) | 8.36 | ±0.93 |
|  | to commit to being active by cycling or going for a daily walk | 8 | (7-8) | 7.67 | ±0.98 | 8 | (7-8) | 7.55 | ±1.13 | 8.5 | (7.75-9) | 8.08 | ±1.35 | 8 | (7-9) | 7.93 | ±1.00 |
|  | to spread daily activities (e.g. shopping, house-work) over the day | 8 | (7.75-9) | 8.08 | ±1.00 | 6 | (6-8.5) | 6.91 | ±1.58 | 8 | (8-9) | 8.00 | ±1.32 | 7 | (5.5-9) | 6.50 | ±2.10 |
|  | to continue to do physical labour (e.g. helping at the farm, renovating, yard work) | 6.5 | (4.75-7.25) | 5.75 | ±2.60 | 7 | (6-8.5) | 7.00 | ±1.61 | 7.5 | (4.75-8.25) | 6.50 | ±2.64 | 9 | (8-9) | 8.36 | ±0.84 |
| **MCC** | to be able to manage other co-existing conditions (e.g. joint problems, pain, heart conditions etc.) | 8.5 | (7.75-9) | 8.25 | ±0.87 | 8 | (7-8) | 7.55 | ±1.13 | 9 | (8-9) | 8.54 | ±0.72 | 9 | (7-9) | 8.14 | ±1.10 |
| **MS** | to be able to do things without getting short of breath | 7 | (7-8.25) | 7.42 | ±1.08 | 7 | (6.5-8) | 7.18 | ±1.25 | 9 | (8.75-9) | 8.50 | ±1.29 | 9 | (8-9) | 8.36 | ±0.93 |
|  | to have more energy | 7.5 | (7-8.25) | 7.42 | ±1.44 | 7 | (6.5-8) | 7.27 | ±1.27 | 9 | (8-9) | 8.38 | ±1.66 | 8 | (7-9) | 7.36 | ±2.17 |
| **E** | to do voluntary work | 5.5 | (4.5-6.25) | 5.17 | ±2.48 | 5 | (4.5-5.5) | 5.09 | ±1.97 | 5.5 | (4-7) | 5.54 | ±2.52 | 9 | (7.25-9) | 8.14 | ±1.35 |
|  | to work less and have more time off | 5 | (4-6.25) | 4.83 | ±2.59 | 5 | (4-6) | 4.91 | ±1.70 | 4 | (2-6.25) | 4.21 | ±2.41 | 8 | (7-9) | 7.86 | ±1.29 |
| **PE** | to have facilities to exercise in their own home | 7 | (5.75-7.25) | 6.42 | ±1.44 | 6 | (6-8) | 6.82 | ±1.47 | 8 | (6-8) | 6.88 | ±2.29 | 9 | (8-9) | 8.07 | ±1.38 |
|  | to monitor and plan activity according to the weather | 7 | (6-8) | 6.92 | ±1.44 | 6 | (5.5-7) | 6.18 | ±1.25 | 7 | (5.75-8) | 6.54 | ±2.15 | 8.5 | (7-9) | 8.00 | ±1.18 |
| **Non-specific** | to take action when set-backs occur | 7 | (7-9) | 7.25 | ±2.18 | 7 | (5.5-8) | 6.91 | ±1.58 | 8 | (8-9) | 8.17 | ±0.87 | 9 | (8-9) | 8.43 | ±0.85 |
|  | to not sit or lay down for prolonged periods during the day | 8 | (6.75-9) | 7.33 | ±2.39 | 7 | (7-8.5) | 7.73 | ±0.90 | 8 | (7-9) | 7.75 | ±1.80 | 8.5 | (7.25-9) | 8.07 | ±1.21 |
|  | to have more surgical options for treatment | 5 | (3.75-6.25) | 4.83 | ±2.25 | 5 | (1.5-5.5) | 4.18 | ±2.52 | 7 | (5-8) | 6.17 | ±2.70 | 7 | (3.5-8.75) | 6.00 | ±3.06 |
| **Professional support** | to be followed up after acute COPD exacerbations (flare up of symptoms) | 8 | (7.75-9) | 8.17 | ±0.83 | 7 | (7-8) | 7.55 | ±0.93 | 8 | (7.5-9) | 8.04 | ±1.11 | 9 | (9-9) | 8.86 | ±0.36 |
|  | to have a guide for exercises that could be done at home | 8 | (7-9) | 8.00 | ±0.85 | 7 | (6-8) | 6.82 | ±1.72 | 9 | (7.5-9) | 8.22 | ±0.95 | 8 | (7.25-9) | 7.79 | ±1.37 |
|  | to be encouraged by health care providers to participate in daily physical activity and avoid sedentary behaviours | 8 | (8-9) | 8.25 | ±0.62 | 7 | (6.5-8) | 7.18 | ±1.17 | 8 | (7-9) | 7.52 | ±1.83 | 8 | (7-9) | 7.71 | ±1.38 |
|  | to be offered maintenance pulmonary rehabilitation programs | 8 | (7.75-9) | 8.00 | ±1.13 | 6 | (4.5-7.5) | 6.00 | ±1.95 | 8 | (8-9) | 8.22 | ±0.80 | 8 | (7-9) | 7.71 | ±1.38 |
|  | to take part in a supervised physical activity program, which includes strength and fitness training and such things as practicing getting up from sitting to standing | 7 | (6.75-8.25) | 7.33 | ±1.30 | 7 | (5.5-7.5) | 6.27 | ±1.95 | 8 | (7-9) | 7.57 | ±1.78 | 8.5 | (8-9) | 8.07 | ±1.27 |
|  | to be provided a specific program targeted at reducing sedentary behaviour | 7.5 | (7-8.25) | 7.17 | ±1.90 | 6 | (5-7.5) | 6.00 | ±2.00 | 8 | (7-9) | 7.61 | ±1.27 | 9 | (8-9) | 8.14 | ±1.29 |
|  | to take part in a daily modified exercise program | 7 | (6.75-8) | 7.17 | ±0.94 | 6 | (4.5-7.5) | 6.18 | ±1.78 | 8 | (7-8.5) | 7.30 | ±1.96 | 8.5 | (8-9) | 8.43 | ±0.65 |
|  | to participate in interventions which include counselling to increase motivation toward changing behaviours for health (motivational interviewing) | 7 | (6-8) | 7.00 | ±1.54 | 6 | (6-7.5) | 6.73 | ±1.10 | 8 | (6-9) | 7.00 | ±2.22 | 9 | (8-9) | 8.43 | v0.76 |
|  | to be managed and supported long-term by a multidisciplinary team including educated nurses and social workers | 8 | (8-9) | 8.25 | ±0.75 | 6 | (5-7.5) | 6.27 | ±1.95 | 8 | (7-9) | 7.30 | ±2.20 | 7 | (7-8.75) | 7.21 | ±1.72 |
|  | to be provided with reminders, messages and prompts for emotional support | 7 | (5-7.25) | 6.17 | ±1.70 | 7 | (6-8) | 6.91 | ±1.38 | 7 | (5.5-8.5) | 6.74 | ±2.20 | 8.5 | (8-9) | 8.29 | ±0.83 |
| **SM + goal setting** | to be provided with an individualised plan and goals to integrate daily activities into their life | 7.5 | (7-8) | 7.42 | ±0.90 | 7 | (6-8) | 7.09 | ±1.51 | 8 | (7-9) | 7.83 | ±1.27 | 9 | (8-9) | 8.50 | ±0.76 |
|  | to set a time limit for sedentary behaviours | 7 | (6-8) | 7.08 | ±1.16 | 7 | (6.5-8) | 7.09 | ±1.14 | 7 | (6-8.5) | 7.09 | ±1.65 | 8 | (7.25-8.75) | 7.71 | ±1.27 |
| **Social support** | to have social interactions with friends and families | 7 | (6.5-8.25) | 6.83 | ±2.12 | 8 | (7-8.5) | 7.27 | ±2.15 | 9 | (7-9) | 7.96 | ±1.55 | 8.5 | (8-9) | 8.21 | ±0.97 |
|  | to have a good social support network and actively involve family and friends in their care | 8 | (6.5-9) | 7.08 | ±2.27 | 8 | (6.5-8.5) | 7.36 | ±1.80 | 8 | (7-9) | 7.52 | ±1.81 | 9 | (8.25-9) | 8.43 | ±1.09 |
|  | to belong to groups, including groups that gets together to exercise | 7 | (5.75-8.25) | 6.58 | ±2.11 | 8 | (5.5-8) | 6.82 | ±2.14 | 8 | (7-9) | 7.35 | ±2.01 | 8 | (7-9) | 7.71 | ±1.33 |
|  | to be helped to build a social life and strengthen social ties | 7 | (4.75-8) | 6.33 | ±2.10 | 7 | (6-8) | 7.09 | ±1.51 | 8 | (6-8.5) | 6.96 | ±2.10 | 8.5 | (7.25-9) | 8.21 | ±0.89 |
|  | to have peer support such as from group programs in local community centres | 7 | (5.75-8) | 6.50 | ±2.24 | 7 | (6-8) | 6.55 | ±2.11 | 7 | (5-8) | 6.30 | ±2.51 | 9 | (8.25-9) | 8.57 | ±0.85 |
|  | to be able to do physical activities together with other patients (e.g. cycling or walking) | 7 | (4.75-8.25) | 6.42 | ±2.39 | 7 | (5.5-8) | 6.64 | ±1.75 | 7 | (5-8) | 6.43 | ±2.46 | 8 | (7.25-8.75) | 7.79 | ±1.19 |
| **Understand patient CFE** | to have their breathlessness and fear of breathlessness treated | 8.5 | (7-9) | 8.08 | ±1.08 | 7 | (6-8) | 7.00 | ±1.41 | 9 | (8-9) | 8.52 | ±0.79 | 8 | (8-9) | 8.14 | ±0.86 |
|  | to be less breathless | 8 | (6.75-9) | 7.42 | ±2.07 | 7 | (5.5-8) | 6.55 | ±1.44 | 9 | (9-9) | 8.70 | ±0.76 | 8 | (6.25-8.75) | 7.36 | ±1.69 |
|  | to be provided with reasons and motivation to not be inactive | 8 | (7-8.25) | 7.75 | ±0.97 | 7 | (6-8) | 7.00 | ±1.18 | 8 | (7.5-9) | 7.83 | ±1.61 | 8 | (7-8.75) | 7.36 | ±1.65 |
| CFE: concerns / fears / expectations; COPD: chronic obstructive pulmonary disease; E: Modify / understand employment commitments; MCC: Manage co-existing problems and conditions; MS: manage symptoms; PA: physical activity; PE: Manage / understand the physical environment; SE: self-efficacy; SM: Self-monitoring  Results from 9-point Likert scale where 1 = unimportant and 9 = very important | | | | | | | | | | | | | | | | | |

| **Table S3:** What is important to improve sleep quality - Group average Likert-score for each item by theme | | | | | | | | | | | | | | | | | |
| --- | --- | --- | --- | --- | --- | --- | --- | --- | --- | --- | --- | --- | --- | --- | --- | --- | --- |
| **Theme** | **Item** | **COPD-E** | | | | **Non-COPD-E** | | | | **SA-COPD** | | | | **NL-COPD** | | | |
|  |  | Median (IQR) | | Mean (±SD) | | Median (IQR) | | Mean (±SD) | | Median (IQR) | | Mean (±SD) | | Median (IQR) | | Mean (±SD) | |
| **Follow sleep hygiene principles** | to be educated on and follow sleep hygiene principles, including going to bed and getting up at the same time every day, avoiding napping during the day, knowing when and what to eat (balanced diet) and avoiding smoking before bed | 8 | (6.75-8) | 7.17 | ±1.59 | 8 | (7-8.5) | 7.73 | ±1.01 | 7 | (7-8) | 7.13 | ±2.12 | 9 | (8-9) | 8.50 | ±0.65 |
|  | to be educated on and follow sleep hygiene principles, including creating a dark, silent and comfortable sleeping environment | 8 | (6.75-8) | 7.00 | ±2.09 | 7 | (6.5-8) | 7.36 | ±1.12 | 7 | (7-8) | 7.22 | ±1.81 | 9 | (8-9) | 8.57 | ±0.51 |
|  | to have a well ventilated house (i.e. good air flow) | 6 | (6-8) | 6.75 | ±1.36 | 7 | (7-8) | 7.09 | ±1.22 | 8 | (6.5-9) | 7.39 | ±1.97 | 8 | (7.25-9) | 7.86 | ±1.35 |
|  | to follow sleep hygiene principles, including removing electronic devices from bedrooms and avoiding screen time one hour before bed | 7 | (4.5-8) | 6.00 | ±2.41 | 7 | (7-8) | 7.45 | ±0.93 | 6 | (4-7.5) | 5.57 | ±2.76 | 8.5 | (8-9) | 8.21 | ±0.97 |
|  | to sleep with two cushions/pillows so the head is lifted | 5.5 | (3.75-7) | 4.92 | ±2.23 | 6 | (5-6.5) | 6.09 | ±1.45 | 7 | (5.5-8.5) | 6.74 | ±2.09 | 8.5 | (8-9) | 7.93 | ±1.49 |
|  | and their families to have their sleep habits discussed | 7.5 | (6-8) | 6.42 | ±2.75 | 7 | (6-7.5) | 6.82 | ±1.40 | 7 | (2.5-8) | 5.52 | ±3.07 | 9 | (7.25-9) | 8.07 | ±1.44 |
|  | to avoid alcohol intake before going to bed | 6 | (5.75-8) | 6.08 | ±1.98 | 7 | (6-7.5) | 6.91 | ±1.14 | 7 | (4-8) | 5.87 | ±2.97 | 8 | (5.5-9) | 7.14 | ±2.03 |
| **Increase PA / fitness** | to be encouraged to increase their physical activity levels and keep active throughout the day; including keeping involved in house-hold duties (cooking, doing the groceries, cleaning etc.) | 7.5 | (5.75-8) | 6.92 | ±1.83 | 8 | (7-9) | 7.91 | ±1.04 | 9 | (8-9) | 8.43 | ±0.66 | 8 | (7.25-9) | 7.86 | ±1.35 |
|  | to be more active during the day so they are tired at night | 7 | (5-8) | 6.42 | ±1.83 | 7 | (7-9) | 7.64 | ±1.21 | 8 | (8-9) | 8.09 | ±1.04 | 8.5 | (8-9) | 8.21 | ±0.97 |
| **Manage anxiety / stress / worry** | to manage anxiety, stress, worry and intrusive thoughts and be able to relax at night | 7.5 | (7-9) | 7.67 | ±1.37 | 7 | (7-8.5) | 7.55 | ±1.21 | 8 | (7.5-9) | 7.91 | ±1.20 | 8.5 | (8-9) | 8.07 | ±1.38 |
|  | to be taught relaxation techniques and skills (e.g. use of meditational music) | 7 | (5.75-8) | 6.75 | ±1.76 | 7 | (7-8.5) | 7.64 | ±1.03 | 8 | (5.5-8.5) | 6.43 | ±2.90 | 7.5 | (7-8.75) | 7.29 | ±1.73 |
|  | to have a partner to reduce anxiety | 7 | (5-7) | 5.83 | ±2.25 | 6 | (5-7) | 6.18 | ±1.25 | 7 | (5-8) | 6.43 | ±2.46 | 8.5 | (7.25-9) | 8.21 | ±0.89 |
| **Manage symptoms** | to build confidence and competence with managing breathlessness | 7 | (6.75-8.25) | 7.17 | ±1.40 | 7 | (6.5-8.5) | 7.36 | ±1.36 | 9 | (8-9) | 8.39 | ±0.78 | 7.5 | (5-8.75) | 7.00 | ±1.84 |
|  | to have symptoms managed that interfere with sleep including cough, breathlessness, pain and having to go to the toilet frequently during the night | 8 | (6.75-9) | 7.50 | ±1.51 | 8 | (6.5-8.5) | 7.64 | ±1.21 | 8 | (7-9) | 7.22 | ±2.37 | 8 | (7-9) | 7.93 | ±1.00 |
|  | to be instructed to take their inhalers before sleep | 7 | (5.75-8) | 6.83 | ±1.34 | 7 | (6-7.5) | 6.73 | ±1.27 | 8 | (6.5-9) | 6.96 | ±2.77 | 8.5 | (8-9) | 8.36 | ±0.74 |
| **NS** | to not sit, lay down or nap for prolonged periods during the day | 6.5 | (5.75-8.25) | 6.83 | ±1.59 | 7 | (6-7.5) | 6.45 | ±1.86 | 8 | (7-9) | 7.61 | ±1.78 | 7 | (7-9) | 7.21 | ±1.97 |
| **SM / GS** | to set goals and self-monitor their sleep | 6.5 | (5.75-7) | 6.17 | ±1.99 | 7 | (6.5-8) | 7.18 | ±0.98 | 8 | (7-8.5) | 7.04 | ±2.53 | 8 | (7-9) | 7.71 | ±1.33 |
| **Understand cause of sleep problem** | to be assessed for the presence of, and treated for, other conditions including heart failure, back pain and digestive symptoms | 8 | (6.75-9) | 7.75 | ±1.42 | 8 | (7-9) | 7.73 | ±1.19 | 8 | (7.5-9) | 7.57 | ±2.29 | 8 | (7.25-9) | 7.43 | ±2.21 |
|  | to be provided non-invasive ventilation (sleep apnoea machine; CPAP) if needed | 8 | (6.5-9) | 7.17 | ±2.44 | 8 | (7.5-8) | 7.73 | ±1.01 | 8 | (6-9) | 6.83 | ±2.92 | 9 | (8-9) | 8.57 | ±0.65 |
|  | to have medications monitored which may impact on sleep | 7.5 | (6.75-9) | 7.50 | ±1.38 | 7 | (7-8) | 7.27 | ±1.10 | 8 | (7.5-9) | 7.30 | ±2.62 | 8 | (7-9) | 7.64 | ±1.50 |
|  | to be referred to a sleep specialist for assessment and treatment of sleep problems | 8 | (6.5-8) | 6.92 | ±2.27 | 7 | (6.5-8) | 7.09 | ±1.04 | 8 | (6.5-9) | 6.74 | ±2.86 | 8.5 | (7-9) | 7.86 | ±1.41 |
|  | To be provided with supplementary oxygen if they have trouble maintaining oxygen levels at night to improve their sleep quality and oxygen levels | 6 | (5-7.5) | 6.00 | ±2.56 | 8 | (7.5-8.5) | 7.73 | ±1.27 | 8 | (6.5-9) | 7.00 | ±2.95 | 8 | (6-9) | 7.43 | ±1.70 |
|  | to lose weight | 6 | (5-7.25) | 6.00 | ±1.95 | 7 | (7-8.5) | 7.45 | ±1.29 | 8 | (5-8.5) | 6.52 | ±2.84 | 8 | (7-9) | 7.86 | ±1.29 |
| CPAP: continuous positive airway pressure; PA: physical activity; NS: Non-specific; SM / GS: Self-monitoring / goal setting  Results from 9-point Likert scale where 1 = unimportant and 9 = very important | | | | | | | | | | | | | | | | | |
