# Supplementary material for: Improving physical activity, sedentary behaviour and sleep in COPD: perspectives of people with COPD and experts via a Delphi approach
Source: PeerJ. 2018 Apr 27;6:e4604. doi: 10.7717/peerj.4604 (PMC5926552; doi:10.7717/peerj.4604)
Supplement: Supplemental Information 1 [file peerj-06-4604-s004.docx]

| **Group** | **Item** |
| --- | --- |
|  | **Unimportant to be more active and participate in more everyday activities** |
| NL-COPD | om een blaasinstrument te spelen [to play a brass instrument] |
| COPD-E | for people with chronic lung disease to be provided with feedback via unobtrusive wearables |
| COPD-E | to use telemedicine, such as remote coaching |
| COPD-E | for people with chronic lung disease to be provided with e-health applications embedded in a self-management program |
| COPD-E | for people with chronic lung disease to monitor the previous weather forecast |
| Non-COPD-E | for people with chronic lung disease to be provided a device/app that tracks activity and rewards them for moving |
| Non-COPD-E | for people with chronic lung disease to be provided with financial incentives |
| Non-COPD-E | for people with chronic lung disease to self-monitor their activity |
| Non-COPD-E | for people with chronic lung disease to be provided a health coach for goal-setting |
| Non-COPD-E | to merge pulmonary rehabilitation programs with cardiac rehabilitation programs |
| Non-COPD-E | for people with chronic lung disease to track their activity |
|  | **Unimportant to spend less time sitting and lying** |
| SA-COPD | for people with chronic lung disease to have a job |
| NL-COPD | om een blaasinstrument te spelen [to play a brass instrument] |
| COPD-E | for people with chronic lung disease to use public transport |
| COPD-E | for people with chronic lung disease to be provided with feed-back through e-health applications |
| COPD-E | for people with chronic lung disease to be provided with a device to remind them to move |
| Non-COPD-E | for people with chronic lung disease to be offered financial incentives |
|  | **Unimportant to improve sleep quality** |
| SA-COPD | for people with chronic lung disease to avoid caffeine intake |
| COPD-E | for people with chronic lung disease to be provided with non-invasive ventilation if they hypoventilate |
| COPD-E | for the pros and cons of improving sleep to be discussed with people with chronic lung disease |
| COPD-E | for people with chronic lung disease to avoid sleeping during the day |
